# Correcting pandemic data analysis through environmental fluid dynamics

**DOI:** 10.1063/5.0055299

**Published:** 2021-06-22

**Authors:** Talib Dbouk, Dimitris Drikakis

**Affiliations:** University of Nicosia, Nicosia CY-2417, Cyprus

## Abstract

It is well established that the data reported for the daily number of infected cases during the first wave of the COVID-19 pandemic were inaccurate, primarily due to insufficient tracing across the populations. Due to the uncertainty of the first wave data mixed with the second wave data, the general conclusions drawn could be misleading. We present an uncertainty quantification model for the infected cases of the pandemic's first wave based on fluid dynamics simulations of the weather effects. The model is physics-based and can rectify a first wave data's inadequacy from a second wave data's adequacy in a pandemic curve. The proposed approach combines environmental seasonality-driven virus transmission rate with pandemic multiwave phenomena to improve statistical predictions' data accuracy. For illustration purposes, we apply the new physics-based model to New York City data.

## I. INTRODUCTION

Modeling and analysis of global epidemiology are very challenging.^1^ The COVID-19 pandemic has been widely spreading due to airborne virus transmission.^2–5^ The coronavirus pandemic has impacted the economics and the environment at a global worldwide scale^6,7^ since March 2020.

The daily number of infected cases, reported during the first wave of the pandemic, was inaccurate due to several factors:

• Insufficient tracing across the population.
• Inaccuracy of testing equipment.^8^
• Lack of reproducible confirmation tests.
• Inaccuracy of management by public and private health institutions.

Many online digital libraries, platforms, and institutions continue to utilize inaccurate data from the first wave. However, statistical comparisons between the first and second wave could lead to misleading conclusions for the reasons mentioned above.

The number of deaths is a more reliable source of information than the number of infected cases. This has been a topic of recent debate.^9,10^ Ioannidis *et al.*^9^ investigated the second vs the first wave of COVID-19 deaths. From available data on the age vs the number of deaths, they correlated the second vs the first wave of COVID-19 deaths to the shifts in age distribution and nursing home fatalities. Graichen^10^ investigated the difference between the first and the second (and third wave) of COVID-19 to form a German and European perspective. Other research works emerge along similar lines. Yue *et al.*^11^ tried to estimate the actual size of a pandemic from surveillance systems. Stamatakis *et al.*^12^ and Huang^13^ investigated pandemic data reported in the United Kingdom to examine associations between lifestyle risk factors and mortality outcomes. The above is due to an unknown lack of representativeness that can affect the magnitude and direction of effect estimates.

We recently showed that two pandemic outbreaks would be inevitable due to environmental weather seasonality.^14^ Our findings were based on high-fidelity, multiphase fluid dynamics, and heat and mass transfer simulations of airborne virus transmission.^15^ We combined the simulation results with epidemiological modeling enhanced by a new airborne infection rate index (AIR) and meteorological data.^14^ It is generally believed that the high-temperature and high-humidity environment is conducive to reducing the transmission rate of the new coronavirus. In our previous research,^14,15^ we showed a complex mechanism associated with the effects of weather conditions on virus transmission. It incorporates combinations of three major parameters: relative humidity, temperature, and wind speed.

The daily infected cases' published data during the first wave is inaccurate and thus can be misleading. We develop a new uncertainty quantification model using environmental-climate data that corrects the daily number of infected cases during the first wave, thus improving the second and first waves' comparative analysis. For illustration purposes, we apply the new model to correct the first wave data reported for NYC between March 2020 and March 2021 for the daily number of infections.

## II. MATHEMATICAL MODEL DEVELOPMENT

The pandemic's second wave data constitute a more reliable source of information than the pandemic's first wave's data recorded starting from early March 2020.

In March 2021, thus after one year's period of the COVID-19 pandemic, we know the following:

• The order of magnitude of the total number of deaths from COVID-19 did not change significantly between the first and the second wave periods.^16^
• The order of magnitude of the number of patients in intensive care units (ICUs) did not change importantly between the first and the second wave periods.^16^

Given the above, one can define an accurate mortality rate, Ψ, in an infected population as the following:

$$\Psi (n)=\beta (n)\times \frac{D_{c}(n)}{I_{c}(n)}.$$ (1)

Moreover, Ψ′(n) could be considered as an inaccurate representation of Ψ(n) which is per unit time and depends on the wavenumber (*n*) of the pandemic such that

$$\Psi \prime (n)=\beta (n)\times \frac{D_{c}(n)}{I_{c}^{\prime }(n)}.$$ (2)

Note that the inaccuracy of Ψ′ is placed only in $I_{c}^{\prime }$. *n *=* *1 and *n *=* *2 denote the first and second wave of the pandemic; *I_c_* and *D_c_* are the stationary cumulative number of infected cases and deaths due to the infection; *β* is the transmission rate of infection per unit time inside a general population *N_p_*. In other words, *β* represents the probability per unit time that a susceptible individual becomes infected.

Liu *et al.* ^17^ shed light on the role of seasonality in the spread of the COVID-19 pandemic. Dbouk and Drikakis^14^ quantified the relationship between the weather seasonality and the transmission rate *β* through extensive fluid mechanics simulations for crucial weather parameters such as temperature, relative humidity and wind. They quantitatively showed how a weather-dependent *β* could produce two pandemic waves during one year. Our analysis and modeling approaches are based on the following premises:

(1) The weather seasonality is a major driving force behind two pandemic waves occurred annually.
(2) The virus fatal strength level did not change importantly between two similar seasons between the first and second wave of the pandemic, i.e., winter 2020 vs winter 2021.
(3) The social behaviors and global restriction strategies did not change significantly between the two waves' periods (i.e., masks, social distance, lockdowns, etc.).
(4) The age-pyramid of a country did not change significantly between the two pandemic waves.

Using the above, the mortality rate among the stationary cumulative number of infected individuals should be approximately the same in two consecutive waves of the pandemic, i.e.,

$$\Psi (n,t)=\Psi (n+1,t+\Delta t_{n})\;\;\;\;\text{if}\;\;\;\;\beta (n,t)=\beta (n+1,t+\Delta t_{n}),$$ (3)

where $\Delta t_{n}=t_{n}^{f}-t_{n}^{i}$ denotes the n^th^-wave period of a pandemic. The final time $t_{n}^{f}$ is determined by

$$\left|\frac{\partial \beta (n)}{\partial t}\right|\leq \varepsilon ,$$ (4)

where *ε* is a positive infinitesimal value. It is worth noting that $\left|\frac{\partial \beta (n)}{\partial t}\right|=\left|\frac{\partial \Psi (n)}{\partial t}\right|$.

It is well established that the non-cumulative number of infected cases I′(1) reported during the first pandemic wave is inaccurate, primarily due to the insufficient tracing across the population. Therefore, we aim to correct the inaccurate (old) data, I′(1), to obtain the more accurate (new) data, *I*(1),

$$\Psi \prime (1)=\beta (1)\times \frac{D(1)}{I\prime (1)}.$$ (5)

Substituting *n *=* *1 in Eq. (3) and using Eqs. (1) and (5) yield

$$I(1)=\frac{\Psi \prime (1)}{\Psi (2)}\times I\prime (1).$$ (6)

## III. NYC CASE

**FIG. 1.**
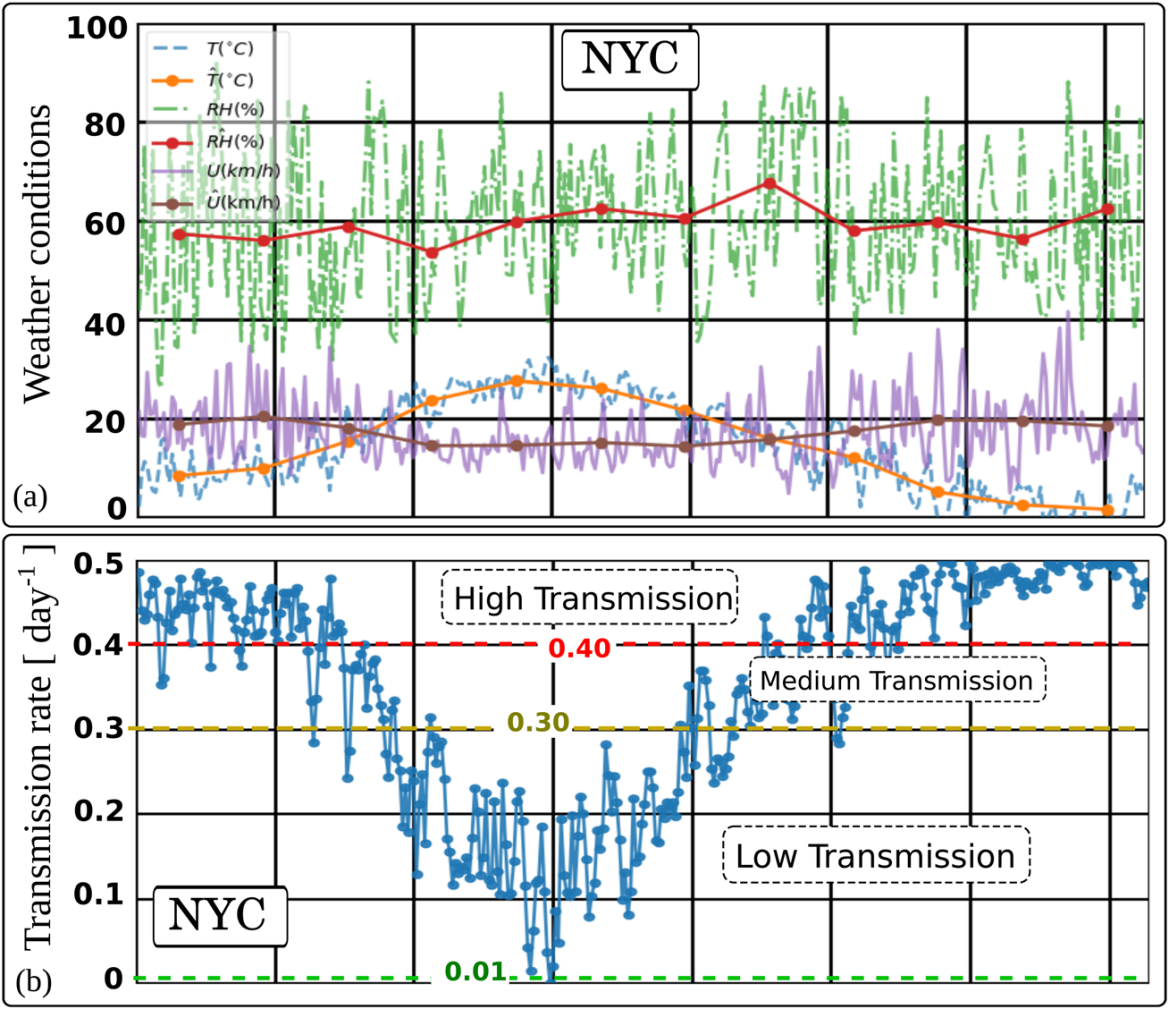
Weather-dependent transmission rate (*β*) in NYC between March 2020 and March 2021 (one-year period). A maximum transmission rate of 0.5 per day means that the probability is P = 1 (100%) for a susceptible individual to be infected in two days ( 1/0.5) due to the weather conditions (wind speed, temperature, and relative humidity) in a region.^14^ (a) NYC weather history data with the hat symbol denoting daily weather data averaged per month. (b) Weather dependent transmission rate (airborne infection rate index ( AIR=β)) showing three trends denoted high, medium, and low separated by the respective threshold values 0.4 and 0.3.

Figure 1 shows the weather-dependent transmission rate (*β*) in NYC between March 2020 and March 2021. A maximum transmission rate of 0.5 per ${\text{day}}^{-1}$, related to the coronavirus airborne concentration rate, means that the probability is P = 1 (100%) for a susceptible individual infected in two days due to the weather conditions (wind speed, temperature, and relative humidity).^14^ Figure 1(a) shows the NYC weather history data with the hat symbol denoting daily weather data averaged per month. Figure 1(b) illustrates the weather-dependent transmission rate (airborne infection rate index ( AIR=β)), showing three trends labeled as high, medium, and low separated by the respective threshold values of 0.4 and 0.3; see Dbouk and Drikakis.^14,15^

**FIG. 2.**
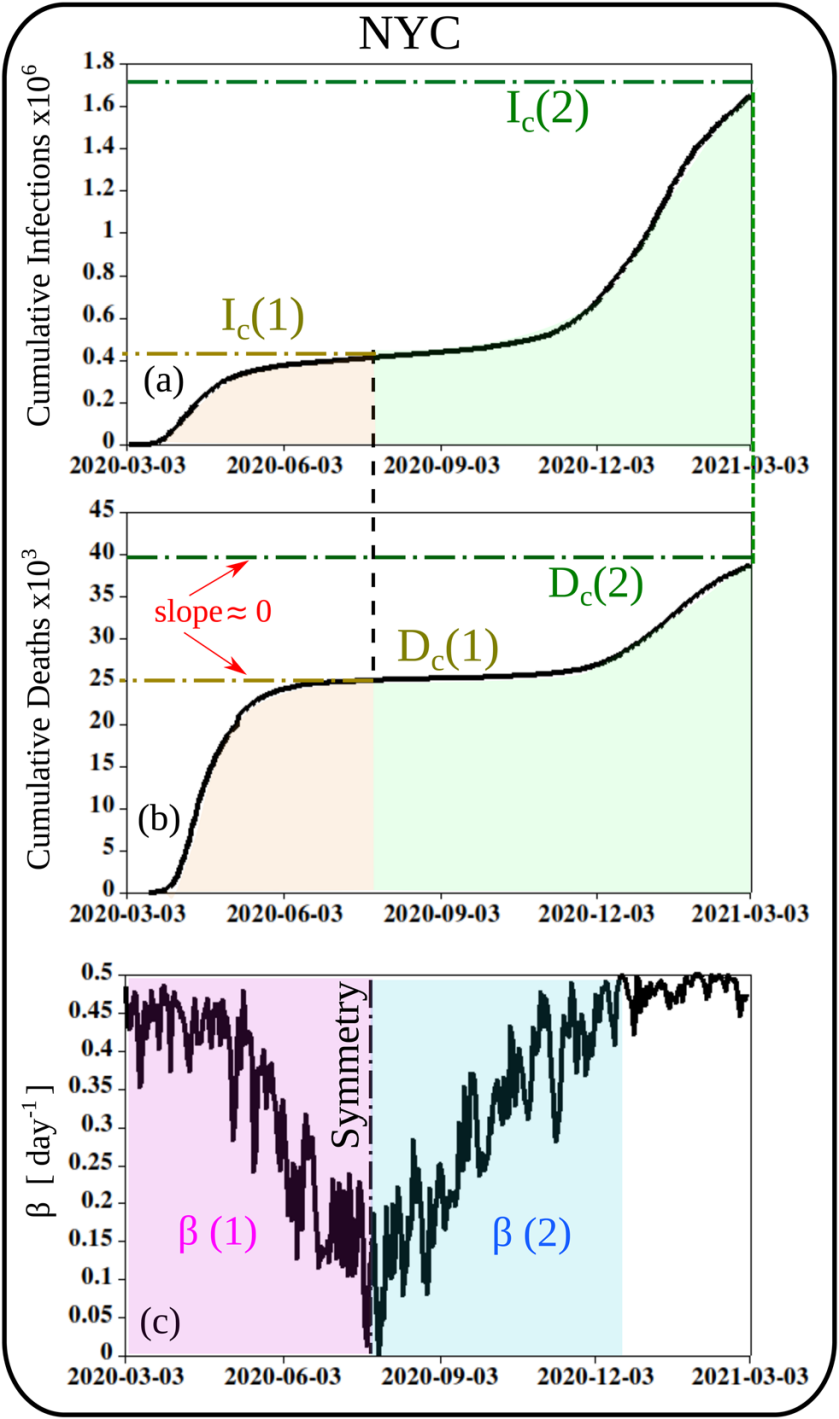
The first (*n *=* *1) and second (*n *=* *2) waves of the COVID-19 pandemic in NYC between March 2020 and March 2021. (a) The cumulative number of infections (*I_c_*); (b) the cumulative number of deaths (*D_c_*); (c) the weather-dependent transmission rate (*β*) as obtained by Dbouk and Drikakis.^14^ β(1) and β(2) are the transmission rate during the first and the second wave of the pandemic, respectively. The pandemic data can be downloaded free of charge from.^18^

The reported pandemic curves data for NYC are shown in Fig. 2 as cumulative number of infections [Fig. 2(a)] and cumulative number of deaths [Fig. 2(b)]. The maximum stationary values for the cumulative infected cases are $I_{c}(1)$ and $I_{c}(2)$ for the first and second waves. Similarly, the cumulative number of deaths is denoted by $D_{c}(1)$ and $D_{c}(2)$ (corresponding to null slopes approximately). Figure 2(c) shows the weather-dependent transmission rate (*β*) as obtained by Dbouk and Drikakis^14^ with β(1) and β(2) representing the transmission rate during the first and the second wave of the pandemic, respectively. This pandemic data can be downloaded free of charge from.^18^ It can be observed from Fig. 2(c) that the symmetry line is well aligned with the maximum stationary values of the cumulative number of deaths ( $D_{c}(1)$) and with the maximum cumulative number of infections ( $I_{c}(1)$). This sheds light on the physics-based behavior of the computed transmission rate [*β* of Fig. 2(c)] driven by the force of weather seasonality in NYC.^14^

**FIG. 3.**
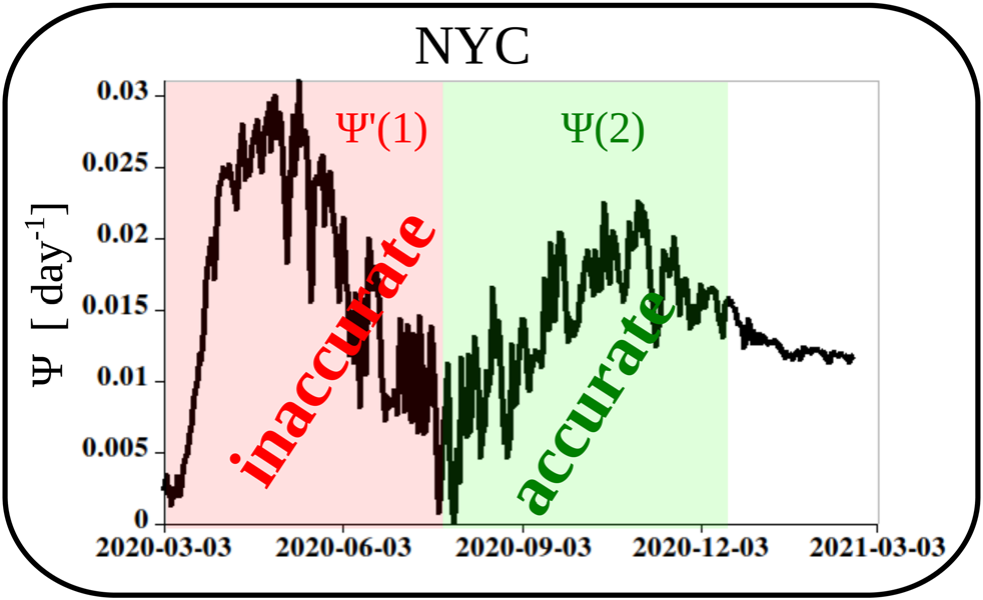
The mortality rate Ψ, per unit time, in the infected population of NYC between March 2020 and March 2021 showing the two waves of the pandemic. An inaccurate Ψ′(1) is observed different from an accurate Ψ(2). This asymmetry between Ψ′(1) and Ψ(2) is a measure of uncertainty quantification of the first wave data's inadequacy concerning the second wave data's adequacy. The daily number of infected cases I′(1) reported during the first pandemic wave also needs to be corrected according to Ψ(1)≈Ψ(2).

Figure 3 shows the mortality rate in the infected population of NYC between 03 March 2020 and 03 March 2021, computed using Eq. (1) with *n *=* *1, and Eq. (2) with *n *=* *2. If both waves data *were* accurate, we should have Ψ(1)≈Ψ(2). Ψ′(1) is, however, different from Ψ(2). Thus, any asymmetry between Ψ′(1) and Ψ(2) is a measure of uncertainty quantification associated with the first wave. The above prompt to correct the inaccurate daily number of infected cases I′(1) reported during the first pandemic wave.

**FIG. 4.**
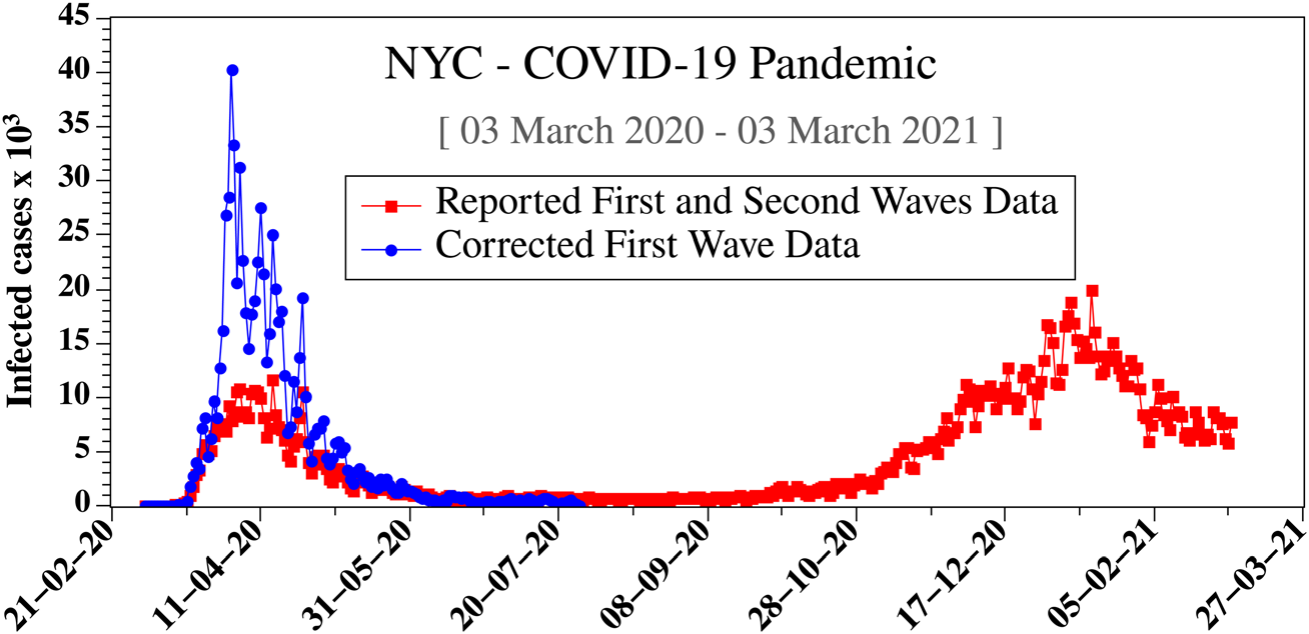
The daily number of COVID-19-infected persons reported for NYC between 03 March 2020 and 03 March 2021 showing the two waves of the pandemic. Squares-line curve: inaccurate reported first wave data I′(1); circles-line curve: corrected first wave data using Eq. (6) for accurate daily number of infected cases *I*(1). The reported pandemic data can be downloaded free of charge from.^18^

The corrected first wave data for the daily number of infected cases in NYC are shown in Fig. 4. One can see that the correction results in increasing the infected cases fourfold.

## IV. CONCLUSIONS AND PERSPECTIVES

The coronavirus pandemic data for the daily number of infections vs the number of deaths during the first wave were incomplete. They lead to misleading conclusions if considered as a data reference. The data inaccuracy for the first wave was primarily due to insufficient tracing across the population. Unfortunately, various online digital libraries and platforms continue to adopt, host, and diffuse these inaccurate data from the first wave, followed by more accurate data of the daily number of infections from the second and subsequent waves.

We proposed a new fluid dynamics, physics-based uncertainty quantification, and correction model that rectifies the first wave data's inadequacy. As an illustration example, we applied the new model to correct the pandemic's first wave data for the daily number of infected cases reported in NYC, USA. The proposed model is limited to regions that witnessed more than one pandemic wave. It can be used to correct their first wave data reported for the daily number of infected cases.

Environmental temperature and humidity affect the ability of the virus to infect, but they are not themselves the decisive factor in preventing the spread of the virus. We cannot rely on seasonal temperature rises to suppress the epidemic. Instead, we should focus more on the formulation and implementation of active epidemic prevention control policies. Social protective measures such as social distancing and face masks will remain important during a pandemic.

## Data Availability

The data that support the findings of this study are available on request from the authors.

## APPENDIX A: MATHEMATICAL DERVATION

The reader can find the computational models and the associated assumptions in previous studies published by the authors.^3,14^ For the carrier bulk multiphase fluid mixture, we have employed the compressible multiphase mixture Reynolds-averaged Navier–Stokes equations in conjunction with the k−ω turbulence model in the shear-stress-transport formulation. The governing equations are detailed in many textbooks.^19,20^

The models accounted for the concentration variation in saliva and predicted airborne virus concentration in expelled saliva droplets under different environmental conditions. From hundreds of computational fluid dynamics simulations, we developed a reduced-order model (ROM) as a new virus airborne infection rate (AIR) index that is directly proportional to the virus concentration rate (CR). AIR is employed to quantify the potential of airborne coronavirus survival under different climate conditions (average temperature, relative humidity, and wind speed) in several worldwide cities.

### 1. Airborne virus particles in saliva: Concentration Rate

For an initial uniform distribution of virus particles, the concentration, *C*, decreases in each airborne saliva droplet as a function of time at different proportions and different rates such that

$$C/C_{0}=e^{-7.5\sqrt{D_{v}(t)\;t}/d_{p}(t)},$$
(A1)

where $d_{p}(t)$ is the saliva droplet diameter that varies with time depending on the evaporation rate and the saliva droplets size distribution. $D_{v}(t)$ is the time-dependent diffusion coefficient of a virus particle in a saliva droplet given by $D_{v}(t)=k_{B}T(t)/3\pi \mu (t)d_{v}$. *d_v_* is the virus capsid external mean diameter (*d_v_* = 100 nm), *T*(*t*) the time-dependent effective temperature of the saliva droplet, *k_B_* the Lattice Boltzmann constant, and μ(t) the time-dependent liquid saliva viscosity.

A positive concentration rate (CR) is defined as follows:

$$CR=\partial (C/C_{0})/\partial t+0.5.$$
(A2)

### 2. Airborne Infection Rate

The CR is directly proportional to the virus survivability. It provides an appropriate indicator for the airborne transmission, which is defined as an “Airborne Infection Rate (AIR)”:

AIR=CR.
(A3)

The CR values between 0 and 0.5 are bounded between 0 and 1 using the operator ⟨*⟩, which transforms a dimensional physical variable *ξ* into a dimensionless one denoted by $\xi^{*}$ such that

$$\xi^{*}=\frac{\xi -\min(\xi )}{\max(\xi )-\min(\xi )},$$
(A4)

where min and max are the minimum and maximum values of *ξ*, respectively.

Many results are obtained for $CR^{*}$ at different weather conditions from several advanced computational fluid dynamics multiphase simulations.^14^ Then, all the data points are well fitted as a function of the relative humidity (RH), the wind speed (U), and the temperature (T),

$$CR^{*}=F\cdot (RH^{*}+U^{*})\cdot sin^{*}(T^{*})+cos^{*}(T^{*}),$$
(A5)

where F is given by

$$F=0.125\left(1-{\left(2T^{*}-1\right)}^{2}\right).$$
(A6)

Note that $CR^{*}$ can be transformed back into *CR* using Eq. (A4) with min(CR)=0, max(CR)=0.5.

In the Subsection,^2^ we will show how CR can be incorporated into epidemiology models, e.g., through a weather-dependent transmission rate *β*.

### 3. Weather-dependent epidemiological model

The extensive high-fidelity simulations led to *CR* = *AIR* as a function of T, RH, and U. We consider AIR as a good indicator for airborne virus transmission and proposed it as a flow physics relevant parameter in epidemiological models.^14^

As a physics-based simulation model, we considered the standard a standard SIR model^21^ given by

$$\frac{dS}{dt}=-\beta \;S\;I/N,$$
(A7)

$$\frac{dI}{dt}=\beta \;S\;I/N-\gamma I,$$
(A8)

$$\frac{dR}{dt}=\gamma \;I.$$
(A.9)

β=AIR is a physics-based weather-dependent parameter transmission rate, and *γ* is the recovery rate coefficient that depends on the individual's health and immunity system. The  beta and *γ* parameters represent the probability per unit time that a susceptible individual becomes infected and the probability per unit time that an infected person becomes recovered and immunized. *t* is time, and *N* is the population number. *S*, *I*, and *R* are the number of *susceptible*, *infected*, and *recovered* individuals, respectively. *β* represents the probability per unit time that a susceptible individual becomes infected. *γ* represents the probability per unit time that an infected person becomes recovered and immune.
